# Triptolide Induces S Phase Arrest and Apoptosis in Gallbladder Cancer Cells

**DOI:** 10.3390/molecules19022612

**Published:** 2014-02-24

**Authors:** Yun-Ping Hu, Zhu-Jun Tan, Xiang-Song Wu, Tian-Yu Liu, Lin Jiang, Run-Fa Bao, Yi-Jun Shu, Mao-Lan Li, Hao Weng, Qian Ding, Feng Tao, Ying-Bin Liu

**Affiliations:** 1 Institute of Biliary Tract Disease, Xinhua Hospital, Affiliated to Shanghai Jiao Tong University, School of Medicine, No. 1665 Kongjiang Road, Shanghai 200092, China; 2 Laboratory of General Surgery, Xinhua Hospital, Affiliated to Shanghai Jiao Tong University, School of Medicine, No. 1665 Kongjiang Road, Shanghai 200092, China; 3 Department of General Surgery, Shanghai Jiao Tong University, School of Medicine, No. 1665 Kongjiang Road, Shanghai 200092, China; 4 Gastrointestinal Surgery, Shaoxing People’s Hospital (Shaoxing Hospital of Zhejiang University), No. 568 Zhongxing North Road, Shaoxing 312000, China

**Keywords:** gallbladder cancer cells, triptolide, proliferation, cell cycle, apoptosis

## Abstract

Gallbladder carcinoma is the most common malignancy of the biliary tract, with a very low 5-year survival rate and extremely poor prognosis. Thus, new effective treatments and drugs are urgently needed for the treatment of this malignancy. In this study, for the first time we investigated the effects of triptolide on gallbladder cancer cells and identified the mechanisms underlying its potential anticancer effects. The MTT assay showed that triptolide decreased cell viability in a dose- and time-dependent manner. The results of the colony formation assay indicated that triptolide strongly suppressed colony formation ability in GBC-SD and SGC-996 cells. Flow cytometric analysis revealed that triptolide induced S phase arrest in gallbladder cancer cells. In addition, triptolide induced apoptosis, as shown by the results of annexin V/propidium iodide double-staining and Hoechst 33342 staining. Furthermore, triptolide decreased mitochondrial membrane potential (ΔΨm) in a dose-dependent manner. Finally, western blot analysis of triptolide-treated cells revealed the activation of caspase-3, caspase-9, PARP, and Bcl-2; this result demonstrated that triptolide induced apoptosis in gallbladder cancer cells by regulating apoptosis-related protein expression, and suggests that triptolide may be a promising drug to treat gallbladder carcinoma.

## 1. Introduction

Gallbladder cancer (GBC) is the most commonly diagnosed malignancy of the biliary tract and the fifth most frequently occurring gastrointestinal cancer [1,2,3,4,5,6]. Because of the nonspecific signs and symptoms, early diagnosis of gallbladder carcinoma is very difficult. Therefore, many patients are diagnosed with advanced GBC, a stage at which treatment by resection is not possible [7,8,9]. As a result, the prognosis of advanced GBC is extremely poor [10,11,12,13], and the 5-year survival rate is less than 10%, as shown by different reports [5,14,15]. Moreover, most patients will experience recurrence of the cancer after surgery and will often not receive effective treatment through chemotherapy or radiotherapy [16,17]. Hence, new effective treatments and drugs are urgently needed in order to improve the outcome of patients with advanced GBC.

**Figure 1:**
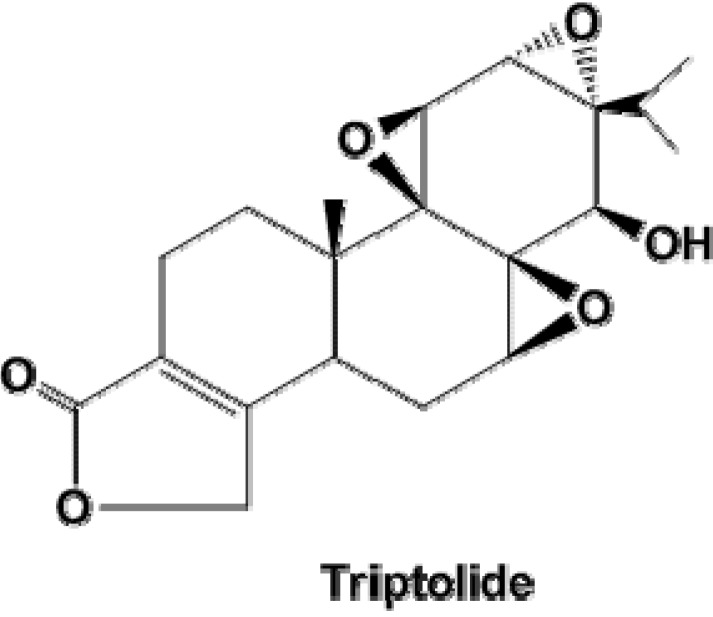
The chemical structure of triptolide.

Triptolide is a small molecule (MW 360.6, Figure 1) isolated from the Chinese Traditional Medicinal herb *Tripterygium wilfordii* Hook F (TWHF). For centuries, TWHF has been used as an effective agent to treat autoimmune diseases such as rheumatoid arthritis, nephritis, and systemic lupus erythematosus [18,19,20,21]. Besides the immunosuppression activity, triptolide has been shown to possess antitumor activity, which has been observed in a wide range of human cancers, including melanoma, breast cancer, lung cancer, bladder cancer, and gastric and colorectal carcinomas [22,23,24,25]. Moreover, triptolide is currently under clinical trials [26]. However, to the best of our knowledge, the effect of triptolide on gallbladder cancer cells and the underlying mechanisms have not been previously investigated. In this study, we report the antineoplastic activity of triptolide in gallbladder cancer cell lines (including GBC-SD and SGC-996 cell lines) and the potential molecular mechanisms underlying this activity. Our results suggest that triptolide may be a promising agent to treat gallbladder carcinoma.

**Figure 2:**
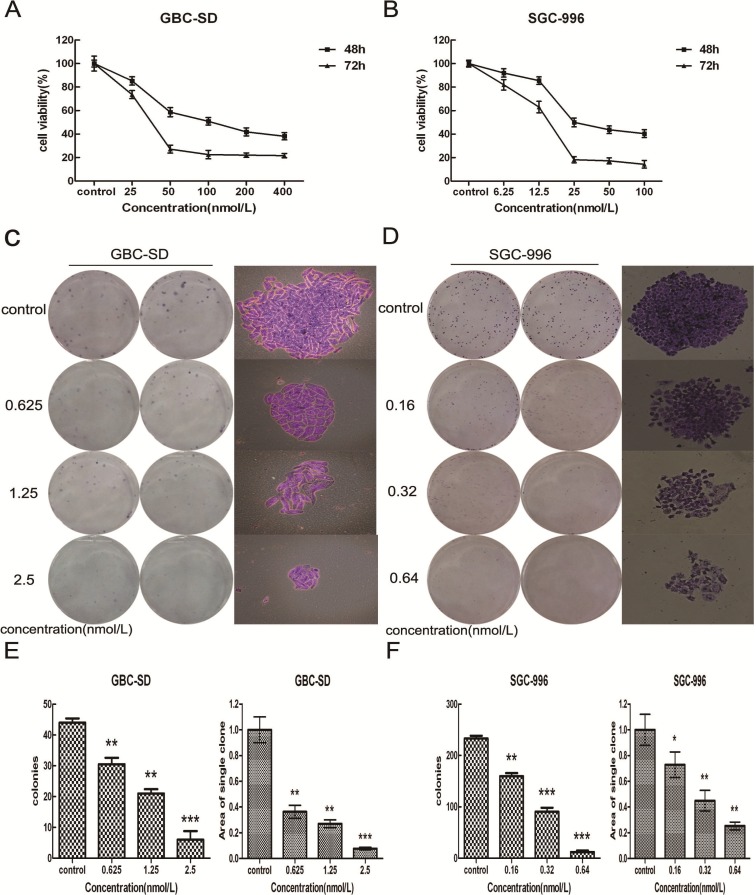
Triptolide dose-dependently decreases proliferation and viability of gallbladder cancer cells. **(A)** GBC-SD cells and (**B)** SGC-996 cells were treated with different concentrations of triptolide for 48 and 72 h. Cell viability and IC50 were determined by MTT assay. **C**–**F** Triptolide suppressed colony formation of GBC-SD and SGC-996 cells. GBC-SD cells and SGC-996 cells were treated with triptolide for 48 h then cells were cultured in fresh medium for 14 days to form colonies. Representative Giemsa staining pictures of the colonies of GBC-SD cells (**C**) and SGC-996 cells (**D**). The number of the colonies of GBC-SD cells (**E**) and SGC-996 cells (**F**) were counted. Data shows the mean ± SD for three independent experiments. * *p* < 0.05; ** *p* < 0.01; *** *p* < 0.001.

## 2. Results and Discussion

### 2.1. Triptolide Decreases Proliferation and Viability of Gallbladder Cancer Cells in a Dose-dependent Manner

To investigate the effects of triptolide on growth and survival of gallbladder cancer cells, we treated GBC-SD and SGC-996 cells with increasing concentrations of triptolide (0–400 nmol/L for GBC-SD, 0–100 nmol/L for SGC-996) for 48 and 72 h. As shown in Figure 2A, B, triptolide induced a time- and dose-dependent decrease in cell viability of GBC-SD and SGC-996 cells. The IC_50_ (the concentration of drug inhibiting 50% of cell growth) of triptolide for GBC-SD and SGC-996 cells at 48 h was approximately 100 nmol/L and 25 nmol/L, respectively. In addition, we investigated the anti-proliferation effect of triptolide on gallbladder cancer cells using the plate-well colony formation assay. As shown in Figure 2C, D, the numbers of colonies of GBC-SD and SGC-996 cells were significantly reduced in a concentration-dependent manner after exposure to triptolide. Moreover, statistical analysis demonstrated that the mean sizes of the triptolide-treated colonies were smaller than those of the control colonies (Figure 2E, F). These data showed that triptolide could inhibit the proliferation ability of gallbladder cancer cells.

The colony formation assay is an effective method for the determination of single cell proliferation capacity, and its basic principle is that a single cell continues proliferation for more than six generations *in vitro*, then becomes a clone which contains more than 50 cells. Usually cells are seeded onto 6-well plates at a density of 200 cells/mL, at this time a very small impact will produce an extremely big effect for cloning. If we choose the same concentration as in the MTT assay in this experiment, then the treated groups cannot form a clone, so we chose concentrations around 10% of the IC_50_ in the colony formation assay.

### 2.2. Triptolide Induces S Phase Arrest and Regulates the Expression of Cell Cycle-related Proteins in GBC-SD and SGC-996 Cells

**Figure 3:**
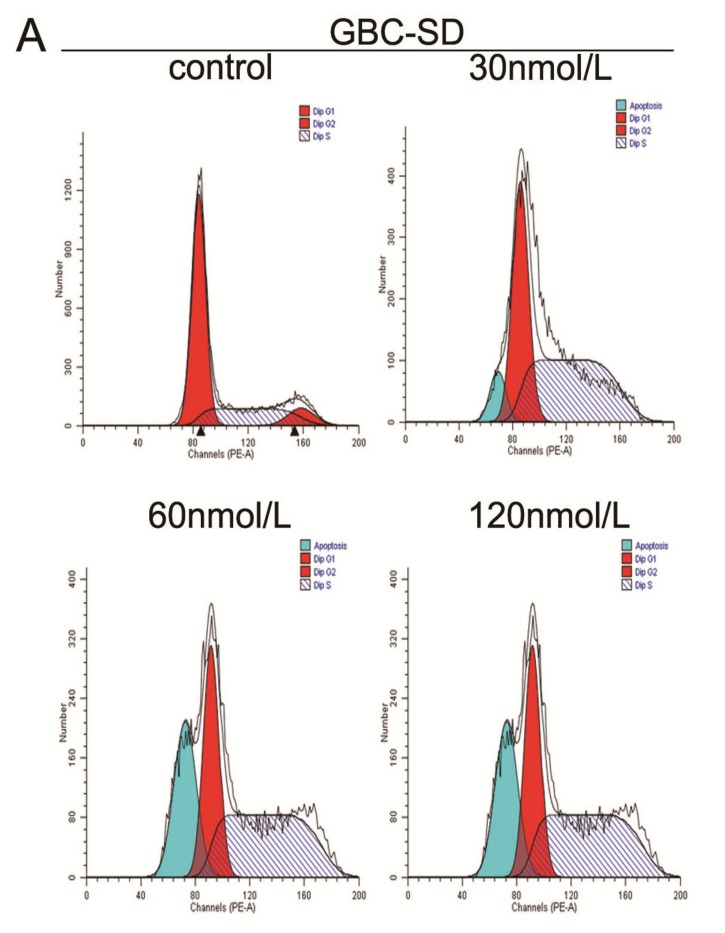

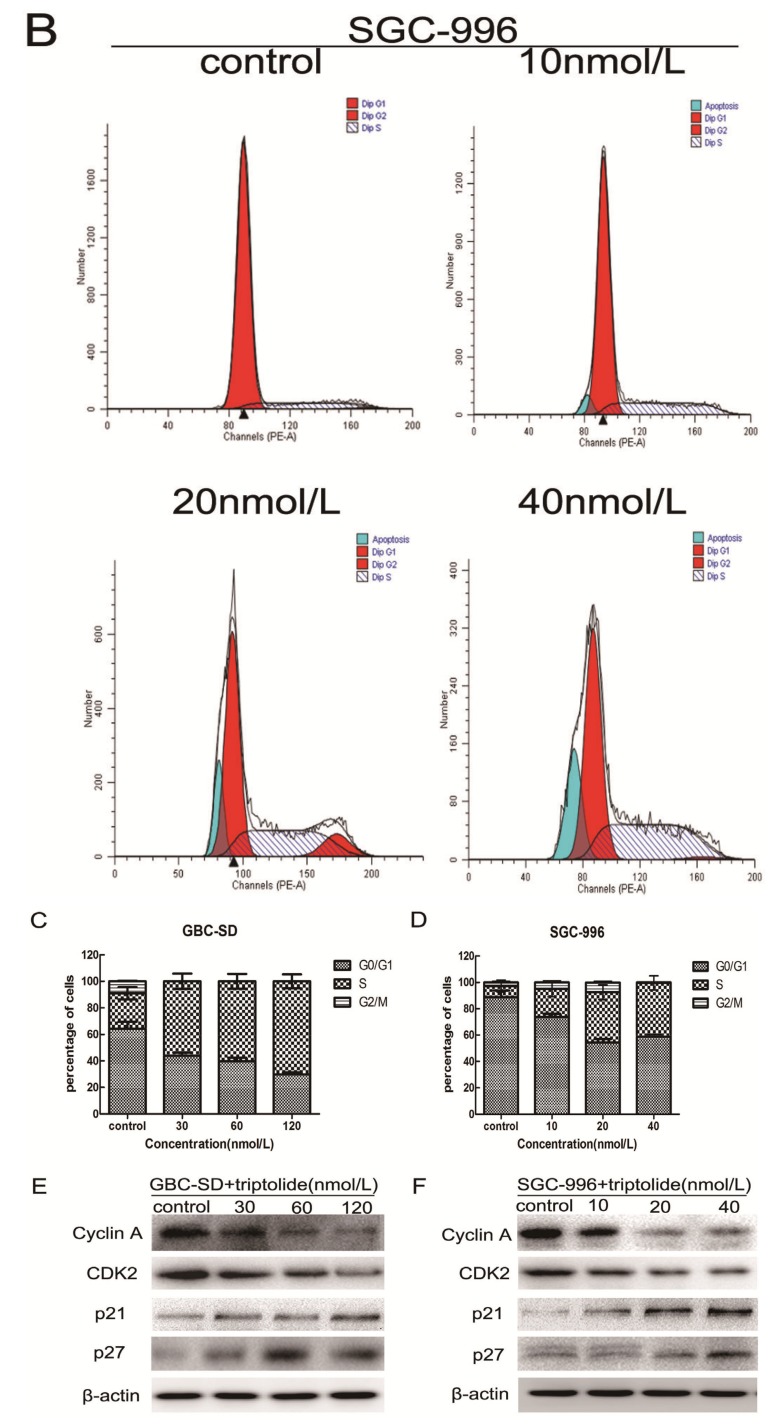
Effect of triptolide on cell cycle distribution in gallbladder cancer cells. (**A**) and (**B**), GBC-SD and SGC-996 cells were treated with various concentrations of triptolide for 48 h, the DNA content was analyzed by flow cytometry. (**C**) and (**D**), The percentage of cells in the G0/G1, S, and G2/M phases of the cell cycle were calculated. Results are expressed as mean ± SD from three independent experiments. (**E** )and (**F**), Effects of triptolide on the protein levels of Cyclin A, CDK2, p21 and p27 in GBC and SGC-996 cells. After incubation with varying concentrations of triptolide for 48 h, whole cell lysates were prepared and western blot analysis was performed using anti-Cyclin A, anti-CDK2, anti-p21 and anti-p27 antibodies, with β-actin serving as a loading control.

To examine the reason for triptolide-mediated cell growth inhibition, cell cycle distribution was analyzed after triptolide treatment using flow cytometry. The results showed that triptolide significantly inhibited cell cycle progression in GBC-SD and SGC-996 cells at 48 h (Figure 3A–D), resulting in a significant increase in the percentage of cells in the S phase( 56.04% ± 5.84, 60.28% ± 5.57% and 70.28% ± 5.28% *vs.* 26.68% ± 4.23% in the control group in GBC-SD cells, *p* < 0.05; 21.3% ± 5.24%, 38.19% ± 5.48% and 40.96 ± 4.89% *vs.* 8.56% ± 2.13% in the control group in SGC-996 cells, *p* < 0.05). In addition, the results for the sub-G1 group (blue color) indicated that triptolide induced apoptosis of gallbladder cancer cells.

Next, we determined the effects of triptolide on cell cycle-related proteins. Cyclins and cyclin-dependent kinases (CDKs) are the two key classes of cell cycle regulatory molecules [27]. Indeed, we observed a dose-dependent decrease in the protein expression of Cyclin A in both gallbladder cancer cell lines after triptolide treatment (Figure 3E, F). Furthermore, triptolide treatment resulted in the downregulation of CDK2 expression and the upregulation of the CDK inhibitors p21 and p27 in both GBC-SD and SGC-996 cells (Figure 3E, F). This finding is consistent with the S phase arrest induced by triptolide. These results indicate that triptolide induces S phase arrest by regulating S phase-related protein expression in gallbladder cancer cells.

### 2.3. Triptolide Induces Apoptosis in Human Gallbladder Cancer Cells

**Figure 4:**
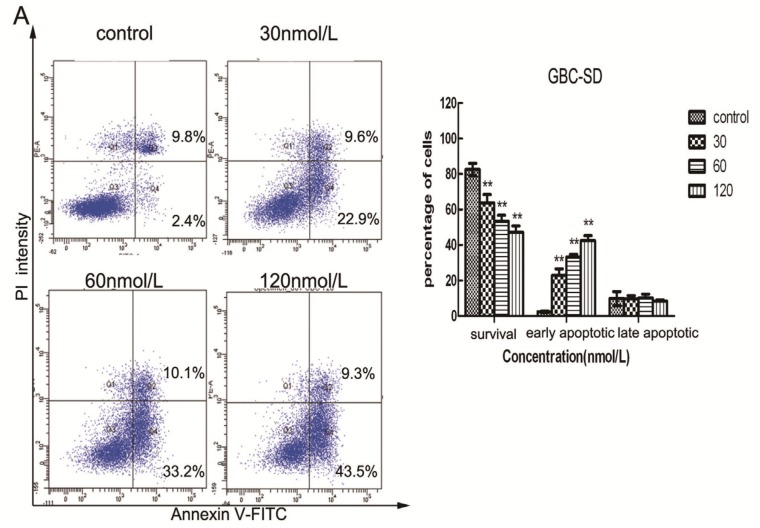

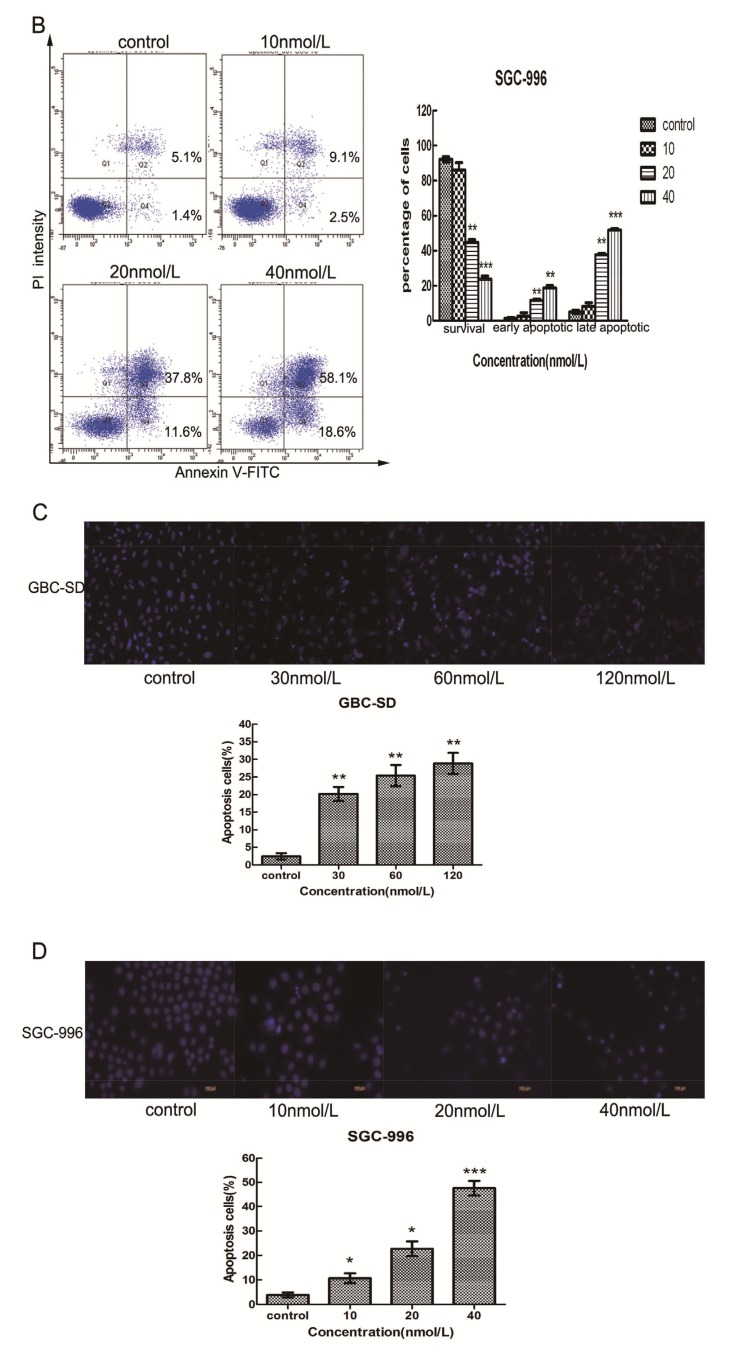
Triptolide induces apoptosis in GBC-SD and SGC-996 cells. GBC-SD cells (**A**) and SGC-996 cells (**B**) were exposed to various concentrations of triptolide for 48 h, then cells were stained with Annexin-V/propidium iodide (PI). The Q3 quadrant (Annexin-V−/PI−), Q4 quadrant (Annexin-V+/PI−), and Q2 quadrant (Annexin-V+/PI+) represent the group of normal cells, early apoptosis, and late apoptosis, respectively. Percentage of surviving cells and cells in early and late apoptosis are shown (**A** and **B**, right panel). The data are the mean ± SD (*n* = 3). * *p* <0.05, ** *p* <0.01, or *** *p* <0.001, compared with the control. (**C**) and (**D**), Apoptotic nuclear morphology changes were assessed by Hoechst 33342 staining with increasing triptolide treatment for 48 h in GBC-SD and SGC-996 cells. Apoptotic cells exhibited highly condensed and fragmented nucleus morphology.

To further confirm whether triptolide induces apoptosis in GBC-SD and SGC-996 cells, the cells were exposed to different concentrations of triptolide for 48 h and then apoptosis was analyzed using annexin V-FITC/PI double staining and flow cytometry. Phosphatidyl serine (PS) is located on the cytoplasmic surface of the cell membrane in normal, live cells. However, in apoptotic cells, PS is translocated from the inner to the outer leaflet of the plasma membrane, thus exposing PS to the external environment. Annexin V-FITC binds to exposed PS on apoptotic and necrotic cells, and PI gains entry into late apoptotic cells and necrotic cells but into not early apoptotic cells and living cells. Cells in the Q3 quadrant (FITC-/PI−) were living, whereas those in the Q2 (FITC+/PI+) and Q4 (FITC+/PI−) quadrants were late and early apoptotic cells, respectively. As shown in Figure 4A, B, triptolide reduced the number of surviving cells and increased the number of both early (22.9% ± 3.67%, 33.2% ± 1.43% and 43.5% ± 2.89% *vs.* 2.4% ± 0.42% in the control group in GBC-SD cells, *p* < 0.05; 2.5% ± 0.98%, 11.6% ± 1.72% and 18.6% ± 1.41% *vs.* 1.4% ± 0.42% in the control group in SGC-996 cells, *p* < 0.05) and late apoptotic cells in a dose-dependent manner. Furthermore, we tested cell apoptosis by Hoechst 33342 staining. When GBC-SD and SGC-996 cells were exposed to various concentrations of triptolide for 48 h, the number of apoptotic cells, characterized by condensed and fragmented nuclei, increased with the dose of triptolide (Figure 4C, D).

### 2.4. Triptolide Decreases Mitochondrial Membrane Potential (ΔΨm)

**Figure 5:**
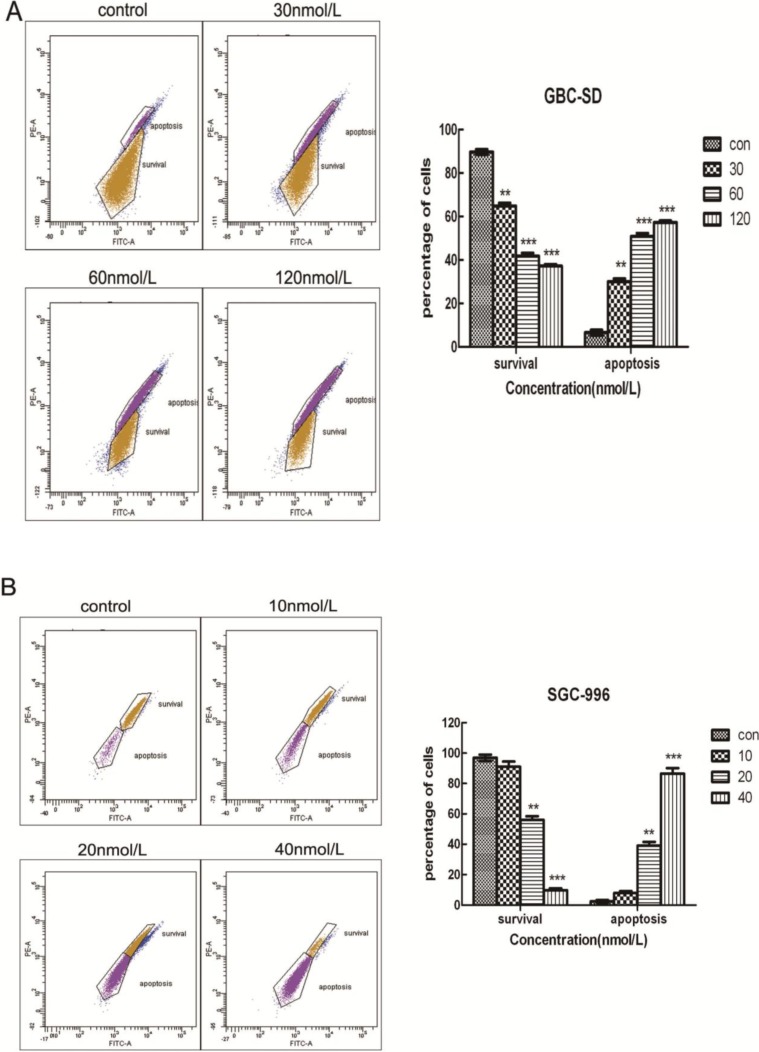
Triptolide decreases mitochondrial membrane potential (ΔΨm) in gallbladder cancer cells. (**A**) and (**B**), Flow cytometric analysis of ΔΨm. GBC-SD and SGC-996 cells were treated with triptolide followed by rhodamine 123 staining. Cells with high ΔΨm are marked “survival” and those with low ΔΨm are marked “apoptosis.” Percentage (%) of cells with high ΔΨm (survival) and low ΔΨm (apoptosis) are shown (**A** and **B**, right panel). Values represent the mean ± SD (*n* = 3). * *p* < 0.05; ** *p* < 0.01; *** *p* < 0.001.

Mitochondria play an important role in the regulation of apoptosis, and apoptosis mediated by the mitochondrial pathway is often associated with the decrease of ΔΨm. To investigate whether mitochondrial membrane integrity is damaged by treatment with triptolide, ΔΨm was examined using rhodamine 123, a yellow-green fluorescent probe for ΔΨm. As shown in Figure 5A, B, triptolide induced a dose-dependent reduction of ΔΨm, as shown by the decrease in the number of surviving cells with normal ΔΨm and the increase in the number of apoptotic cells with low ΔΨm. We found that more than 55% of GBC-SD cells and 85% of SGC-996 cells showed a reduction in ΔΨm after treatment with 120 nmol/L and 40 nmol/L triptolide for 48 h, respectively. It is noteworthy that the percentage of survival cells (for Figure 5A, B) is inconsistent to the survival ratio (for Figure 2A, B), cell cycle distribution (for Figure 3A, B) and PI/Annexin-V staining (for Figure 4A, B). This may be due to the different methods used to detect survival and apoptotic cells or may be caused by different density and status of cells. These results indicated that triptolide induced apoptosis in gallbladder cancer cells via a mitochondria-dependent mechanism.

### 2.5. Triptolide-induced Apoptosis via Regulation of Caspase-3 and Bcl-2 Family Members in Gallbladder Cancer Cells

**Figure 6:**
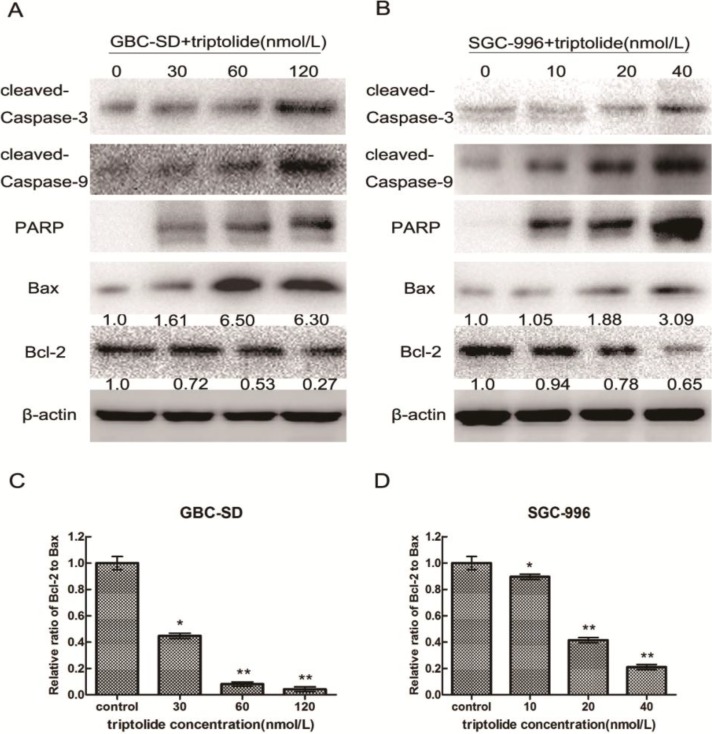
Triptolide regulates the expression of apoptosis-related proteins in gallbladder cancer cells. Expression of cleaved-caspase-3, cleaved-caspase-9, cleaved-PARP, Bax, and Bcl-2 in GBC-SD cells (**A**) and SGC-996 cells (**B**) treated with triptolide at the indicated doses for 48h. β-actin was used as a loading control. (**C**) and (**D**), Ratio of Bcl-2 to Bax determined by band density, shown as mean ± SD, compared with the control (designated as 1.00). * *p* < 0.05; ** *p* < 0.01.

To investigate the underlying molecular mechanism of triptolide-induced apoptosis on GBC-SD and SGC-996 cells, we evaluated the expression of apoptosis-related proteins (cleaved-PARP, cleaved-caspase-3, cleaved-caspas-9, Bax, and Bcl-2) using western blot analysis after treatment of cells with various concentrations of triptolide for 48 h. As shown in Figure 6A, B, treatment with triptolide resulted in the upregulation of Bax, cleaved-caspase-3, cleaved-caspase-9 and cleaved-PARP expression and downregulation of Bcl-2 expression in a dose-dependent manner. Furthermore, we found that the Bcl-2 (antiapoptoic) to Bax (proapoptotic) ratio was significantly lower in the triptolide-treated groups than in the control group (Figure 6C, D). Collectively, the upregulation of Bax, cleaved-caspase-3, cleaved-caspase-9 and cleaved-PARP expression and downregulation of Bcl-2 expression could mediate triptolide-induced apoptosis in gallbladder cancer cells.

The initiation of the apoptosis is controlled by two major pathways: the mitochondria-mediated intrinsic pathway and the death-receptor-induced extrinsic pathway [28]. In the extrinsic pathway, the binding of ligands to membrane death-receptors leads to the activation of caspase-8; in the intrinsic pathway, the release of cytochrome c from the mitochondria results in apoptosome formation and activation of caspase-9 [29]. The activated caspases will then cleave a number of substrates, such as PARP, resulting in the biochemical and morphological changes. In our study, we found that triptolide treatment of GBC-SD and SGC-996 cells activated caspase-9 and caspase-3, accompanied by the increased cleavage of PARP. The activation of caspase-3 usually leads to the cleavage of cytoplasmic (such as PKCδ) and nuclear (such as PARP or lamin) substrates [30,31,32]. As shown in Figure 6A, B, treatment with triptolide resulted in the upregulation of cleaved-caspase-3 and cleaved-PARP expression in a dose-dependent manner. However, we observed some inconsistence between the cleaved-PARP and its upstream regulator cleaved caspase-3, especially in the control group. This may be due to multiple substrates(such as PKCδ or lamin) of cleaved caspase-3 or the low activity of caspase in the control group. In addition, a decrease in ΔΨm was observed after treatment with triptolide in gallbladder cancer cell lines for 48 h. These results indicate that triptolide-induced apoptosis in gallbladder cancer cells might occur via the mitochondrial pathway. Proteins in the Bcl-2 family, including apoptosis-promoting protein Bax and anti-apoptotic protein Bcl-2, play key roles in the mitochondria-mediated apoptosis pathway. The ratio of Bcl-2 to Bax determines the occurrence and severity of apoptosis during apoptotic stimuli. An increased ratio of Bcl-2 to Bax promotes cell survival, whereas a decreased ratio of Bcl-2 to Bax promotes cell apoptosis [33]. Through our western blot assay, we found that that the ratio of Bcl-2 to Bax reduced by 23- and 4.75-fold after the addition of 120 nmol/L and 40 nmol/L triptolide to GBC-SD and SGC-996 cells, respectively, compared with the control group.

## 3. Experimental

### 3.1. Drugs and Antibodies

Triptolide (Sigma-Aldrich, St. Louis, MO, USA) was dissolved in dimethyl sulfoxide (DMSO) to obtain a 1 mM stock concentration and then diluted in the culture medium at various concentrations for different experiments. The primary antibodies used for western blotting were as follows: rabbit anti-Bcl-2, anti-Bax, anti-caspase-3, anti-caspase-9, anti-PARP, anti-Cyclin A, anti-CDK2, anti-p21, anti-p27 and mouse anti-β-actin. All antibodies were purchased from Cell Signaling Technology (Beverly, MA, USA).

### 3.2. Cell Lines and Culture

The human gallbladder cancer cell line GBC-SD was purchased from Shanghai Institute of Cell Biology, Chinese Academy of Sciences (CAS, Shanghai, China), and was grown in high-glucose DMEM (Gibco, Grand Island, NY, USA) supplemented with 10% fetal bovine serum (Gibco), 100 μg/mL streptomycin, and 100 U/mL penicillin (Hyclone, Logan, UT, USA). The other human gallbladder cancer cell line, SGC-996, was obtained from the Medical School at Tongji University (Shanghai, China) and was cultured in RPMI-1640 medium (Gibco) supplemented with 10% FBS and 1% penicillin-streptomycin. Both cell lines were cultured at 37 °C and 5% CO_2_.

### 3.3. Cell Viability Assay

Cell viability was determined as previously described [34]. Briefly, GBC-SD and SGC-996 cells were seeded into 96-well plates at a density of 5,000 cells per well, incubated overnight, and then exposed to various concentrations of triptolide (0, 25, 50, 100, 200 or 400 nmol/L for GBC-SD; 0, 6.25, 12.5, 25, 50, or 100 nmol/L for SGC-996) for 48 and 72 h. Then, 20 μL of 5 mg/mL 3-(4,5-dimethylthiazol-2-yl)-2, 5-diphenyltetrazolium (MTT, Sigma-Aldrich) was added to each well of the plate, and the plates were incubated for 4 h at 37 °C. After removal of the supernatant, 100 μL of DMSO was added to solubilize the formazan crystals. The optical density (OD) was determined at 490 nm using a microplate reader (Bio-Tek, Winooski, VT, USA). The results represent the average of 3 independent experiments. Cell viability was calculated according to the following formula: cell viability = (OD_treatment_/OD_control_) × 100%.

### 3.4. Colony Formation Assay

Cells in the logarithmic growth phase were aliquoted as single cell suspensions and 400 cells were placed into each well of 6-well plates (Corning, Corning, NY, USA). After adherence, cells were treated with triptolide (0.625, 1.25, and 2.5 nmol/L for GBC-SD; 0.16, 0.32, 0.64 nmol/L for SGC-996) for 48 h. Then, all plates were incubated for 2 weeks to allow colony formation. Next, cells were fixed with 4% paraformaldehyde and stained with 0.1% crystal violet (Sigma-Aldrich). After washing, the plates were air-dried, and stained colonies were photographed using a microscope (Leica, Wetzlar, Germany). The total number of colonies (>50 cells/colony) was counted, and the results were expressed as the average of three independent experiments performed over multiple days.

### 3.5. Cell Cycle Analysis

GBC-SD and SGC-996 cells were treated with different concentrations of triptolide for 48 h. Cells were then harvested by trypsinization, washed twice in cold PBS, and fixed in 70% ethanol at 4 °C overnight. After fixation, the cells were washed and resuspended in cold PBS and incubated in a solution of 10 mg/mL RNase and 1 mg/mL propidium iodide (Sigma-Aldrich) at 37 °C for 30 min in the dark. Finally, the DNA content was determined by flow cytometry (BD Biosciences, San Diego, CA, USA). The percentage of cells in the G0/G1, S, and G2/M phases was determined using Cell Quest acquisition software (BD Biosciences).

### 3.6. Cell Apoptosis Assay

Apoptosis was analyzed using the annexin V/propidium iodide apoptosis kit according to the manufacturer’s recommendation (Invitrogen, Carlsbad, CA, USA). Briefly, GBC-SD and SGC-996 cells were seeded in 6-well plates (1 × 10^6^ cells/well) and treated with various concentrations of triptolide for 48 h. The cells were collected, washed with cold PBS, and were resuspended in 100 μL binding buffer at a density of 1 × 10^6^ cells/mL. Then, 5 μL of annexin V-FITC and 5 μL of propidium iodide (PI) working solution (100 μg/mL) were added to 100-μL aliquots of the cell suspension. After incubation for 15 min at room temperature away from light, 400 μL of the binding buffer was added to suspension. The samples were then immediately analyzed by flow cytometry. Each sample was assayed in duplicate, and the experiment was repeated three times.

### 3.7. Hoechst 33342 Staining Assay

Nuclear fragmentation was detected by staining apoptotic nuclei with Hoechst 33342. GBC-SD and SGC-996 cells were treated with different concentrations of triptolide for 48 h, collected and washed, and fixed with acetic acid:ethanol (1:3) for 15 min at room temperature. The fixed cells were stained with 5 μg/mL Hoechst 33342 for 10 min. Digital images were captured using a fluorescence microscope (Leica).

### 3.8. Mitochondrial Membrane Potential (ΔΨm) Assay

Rhodamine 123 (Rho123) was used to determine mitochondrial membrane potential (ΔΨm) as described previously [35]. After treatment with different concentrations of triptolide for 48 h, GBC-SD and SGC-996 cells were harvested and washed twice with cold PBS. The cells were then incubated with rhodamine 123 (Sigma-Aldrich) for 30 min at 5% CO_2_ and 37 °C in the dark. Subsequently, the cells were washed twice with cold PBS and analyzed by flow cytometry.

### 3.9. Western Blot Analysis

GBC-SD and SGC-996 cells were treated with triptolide for 48 h, and whole-cell lysates were prepared using lysis buffer (Beyotime, Shanghai, China) supplemented with Roche Complete Mini Protease Inhibitor Cocktail (Roche, Basel, Switzerland), which was added immediately before the lysis buffer was used. The protein concentration in the cell extracts was determined using the bicinchoninic acid assay system (Beyotime) with BSA as a standard. Equal amounts of protein from each sample were separated by SDS-PAGE and transferred to polyvinylidene difluoride membranes (Millipore, Bedford, MA, USA). Membranes were blocked with 5% skim milk and then incubated with anti-caspase-3, anti-caspase-9, anti-Bcl-2, anti-Cyclin A, anti-CDK2, anti-p21, anti-p27, anti-Bax, anti-PARP, or anti-β-actin antibodies (1:1000; Cell Signaling Technology) at 4 °C overnight. After washing with TBST buffer, the membranes were incubated with a horseradish peroxidase-conjugated goat anti-rabbit/anti-mouse secondary antibody (1:5,000; Abcam, Cambridge, UK), and the bands were visualized using an enhanced chemiluminescent detection reagent from Pierce (Rockford, IL, USA). Photographs were obtained and the bands were scanned, using the Gel Doc 2000 (BioRad, Hercules, CA, USA), to determine the optical densities and quantify the proteins.

### 3.10. Statistical analysis

Statistical analysis was carried out using the SPSS13.0 software. All values are expressed as the mean ± SD unless otherwise stated. Student’s *t*-test was performed to compare the differences between treated groups and their controls. A *p-*value of less than 0.05 (denoted by *) was considered statistically significant (*** *p* < 0.001, ** *p* < 0.01).

## 4. Conclusions

In summary, we have demonstrated that triptolide effectively induces apoptosis in gallbladder cancer cells, probably via the mitochondria-mediated apoptosis pathway. All of the above data indicate that triptolide may be a promising drug to treat gallbladder carcinoma. However, further studies are required to unveil the deep mechanisms underlying the apoptosis and cell cycle arrest induced by triptolide in gallbladder cancer cells.
